# Interpretable Machine Learning Framework to Predict the Glass Transition Temperature of Polymers

**DOI:** 10.3390/polym16081049

**Published:** 2024-04-10

**Authors:** Md. Jamal Uddin, Jitang Fan

**Affiliations:** School of Mechatronical Engineering, Beijing Institute of Technology, Beijing 100081, China

**Keywords:** machine learning, feature selection, hyper-parameter optimization, glass transition temperature, polymer

## Abstract

The glass transition temperature of polymers is a key parameter in meeting the application requirements for energy absorption. Previous studies have provided some data from slow, expensive trial-and-error procedures. By recognizing these data, machine learning algorithms are able to extract valuable knowledge and disclose essential insights. In this study, a dataset of 7174 samples was utilized. The polymers were numerically represented using two methods: Morgan fingerprint and molecular descriptor. During preprocessing, the dataset was scaled using a standard scaler technique. We removed the features with small variance from the dataset and used the Pearson correlation technique to exclude the features that were highly connected. Then, the most significant features were selected using the recursive feature elimination method. Nine machine learning techniques were employed to predict the glass transition temperature and tune their hyperparameters. The models were compared using the performance metrics of mean absolute error (MAE), root mean square error (RMSE), and coefficient of determination (R^2^). We observed that the extra tree regressor provided the best results. Significant features were also identified using statistical machine learning methods. The SHAP method was also employed to demonstrate the influence of each feature on the model’s output. This framework can be adaptable to other properties at a low computational expense.

## 1. Introduction

The term “polymer” encompasses a diverse array of substances that are characterized by the existence of extended molecular chains composed of recurring monomeric units [1,2,3,4,5]. Polymerization is the process through which monomers undergo chemical bonding to form polymer chains. Polymers possess a wide range of properties, including mechanical strength, flexibility, high temperature resistance, and electrical conductivity, which are identified by their distinctive composition and molecular structure [6,7,8,9]. They are extensively employed in several productions such as packaging, automotives, manufacturing, textiles, electronics, medical, and others due to their diverse features. Medical devices, tissue engineering, implants, and other applications use polymers extensively, and the electronic industry uses them in circuit boards, insulation, and packaging. Polymers are also utilized in the automotive industry for applications like tires and bumpers. The rapid synthesis or discovery of new polymers, along with the likelihood of numerous undiscovered polymers, necessitates a comprehensive understanding and documentation of the physical and mechanical features associated with these polymers. One such crucial property is the glass transition temperature of polymers. The glass transition temperature (Tg) is usually characterized as the temperature range at which the polymer makes a shift from a rigid, glass-like state to a more flexible, elastic state [10,11,12,13,14,15,16,17]. The Tg value of a polymer is acknowledged to be affected by the polymer’s chain mobility or volume without chains. The properties of a polymer are decided by its molecular mass, cross-links, side groups, and chain ends. Although theoretical investigations have given crucial insights into the polymer glass transition, they are inadequate for accurate forecasts of polymer Tg and inverse polymer synthesis. The precise estimation of the glass transition temperature is of the utmost importance in customizing polymers to fulfill specific application demands, since it directly impacts their functionality and durability. 

Various techniques can be employed to ascertain the glass transition temperature, including differential scanning calorimetry (DSC), differential thermal analysis (DTA), and thermal mechanical analysis (TMA) [18]. Despite possessing a considerable breadth of knowledge in the field of polymers, the task of creating a polymer with a precise glass transition temperature remains a formidable challenge. Therefore, making a prediction tool for figuring out the glass transition temperature of polymers is very important and helpful in finding new polymers and making new products. Various modeling techniques, including molecular dynamics (MD) and Monte Carlo (MC), have been utilized in the estimation of the glass transition temperature of polymers. Nevertheless, the efficacy of simulations and the computational expenditure pose significant limitations.

Machine learning (ML) [19,20] techniques provide viable alternatives for predicting polymer attributes. This approach expedites the progression of material development and facilitates the execution of intricate computations that are beyond human cognitive capabilities. Machine learning has demonstrated significant effects in various domains, including diverse catalyst design, material design, and drug research. It has found application in the highly efficient screening of polymer qualities due to its ability to bypass the need for expensive computer calculations, such as quantum chemical calculations and molecular dynamics (MD) simulations, which require a deep understanding of chemical properties. Various ML algorithms, including decision trees (DTs), support vector regression (SVR), adaptive boosting (AB), K-nearest neighbors (KNN), random forests (RFs), extreme gradient boosting (XGBoost), hist gradient boosting (HGB), light gradient boosting machines (LGBMs), and extra tree (ET), have been employed to predict the glass transition temperature of polymers and to optimize process conditions.

Several investigations have been conducted for predicting the glass transition temperature through the utilization of various machine learning algorithms. For example, Cassar et al. [21] developed and implemented an artificial neural network with the purpose of generating a predictive model for determining the glass transition temperature of multicomponent oxide glasses. An optimization approach was employed to determine the optimal hyperparameter values employed in an artificial neural network, resulting in the development of an algorithm with superior predictive performance. Then, Alcobaca et al. [22] explored various machine learning techniques that might be employed for the prediction of the glass transition temperature using the chemical composition of the glasses as the input. Additionally, the researchers optimized the hyperparameters of the machine learning algorithms. The findings indicate that the random forest method is the most effective machine learning approach for forecasting Tg.

Zhang et al. [23] made predictions about the glass transition temperature (Tg) by using the molecular traceless quadrupole moment and the molecule average hexadecapole moment parameters as descriptors in a Gaussian process regression model. The investigation encompassed a dataset of 60 samples with Tg values ranging from 194 K to 440 K. The resulting model exhibited favorable attributes, including rapidity, cost-effectiveness, and a high degree of accuracy and stability in estimating Tg. Meanwhile, Yan et al. [24] developed a new machine learning (ML) framework to predict the recovery stresses of thermosetting shape memory polymers (TSMPs). To demonstrate this framework, two new epoxy networks were made and tested, and the ML model was used to figure out the amount of stress that was needed to recover from. Then, Zhang et al. [25] devised the Gaussian process regression (GPR) model for the purpose of forecasting the glass transition temperature of styrenic copolymers. The model demonstrated a high level of accuracy and stability in its prediction. It exhibited a plain and simplistic nature, necessitating a reduced number of parameters in comparison to other alternative modeling techniques.

Lee et al. [26] used extended connectivity fingerprints and traditional QSPR fingerprints to make machine learning models that could accurately predict the glass transition temperature, melting temperature, density, and tensile modulus. The non-linear model using the random forest method was found to be more accurate than linear regression in general. However, using feature selection or regularization, the accuracy of the linear models was shown to be significantly improved. In another study, Tao et al. [27] conducted comprehensive benchmark research involving the compilation of 79 distinct machine learning models, which were subsequently trained on a dataset. Representation is determined based on several features, such as Morgan fingerprinting with or without substructure frequency, RDKit descriptors, molecular embedding, molecular graphs, etc. The combination of the random forest and Morgan fingerprint with frequency (MFF) yielded the most favorable outcomes.

In this study, a comprehensible machine learning framework was devised to forecast the glass transition temperature of polymers. Initially, the Morgan fingerprint and molecular descriptor were employed to quantitatively depict polymer data. Subsequently, the recursive feature elimination technique was utilized to select the most salient features. In order to forecast the glass transition temperature, a comprehensive array of nine machine learning methodologies was utilized, encompassing decision trees, support vector machines, AdaBoost, K-nearest neighbor, extreme gradient boosting, random forests, light gradient boosting, histogram gradient boosting, and extra tree. Furthermore, the hyperparameters of these approaches were also fine-tuned. The attributes were ranked using machine learning and statistical methodologies. The SHAP methodology is employed to demonstrate the influence of specific features on the model’s output. In summary, this study presents the following contributions:

- To evaluate the effectiveness of various machine learning algorithms on polymer data.
- To implement feature representation techniques for polymers.
- To rank and identify significant features associated with polymers.
- To propose an interpretable machine learning framework to predict the glass transition temperature of polymers.
- To implement the SHAP technique to demonstrate the effects of specific features on the model’s output.

The subsequent sections of this paper are structured in the following manner: Section 2 provides a description of the proposed research methodology and outlines the materials utilized in the course of the investigation. Section 3 and Section 4 of this paper encompass the results of the experiments and subsequent analyses. Section 5 provides a summary of our research findings and presents an outline for future directions based on our study.

## 2. Materials and Methods

**Figure 1:**
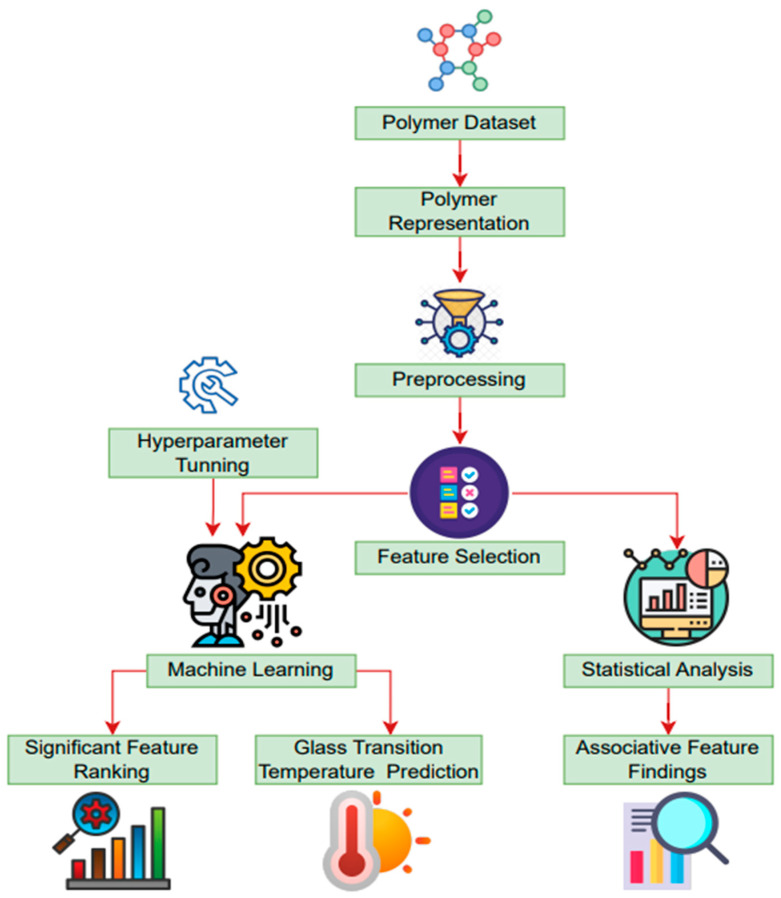
Machine learning framework for predicting polymer glass transition temperature.

In the present study, an open-source polymer dataset was utilized, and a combination of statistical and machine learning methodologies were employed. During the analysis, a series of processes were identified, including data pre-processing, polymer representation, feature selection, parameter tuning, model building, model training, and testing. The workflow of this study is illustrated in Figure 1.

### 2.1. Polymer Dataset

**Figure 2:**
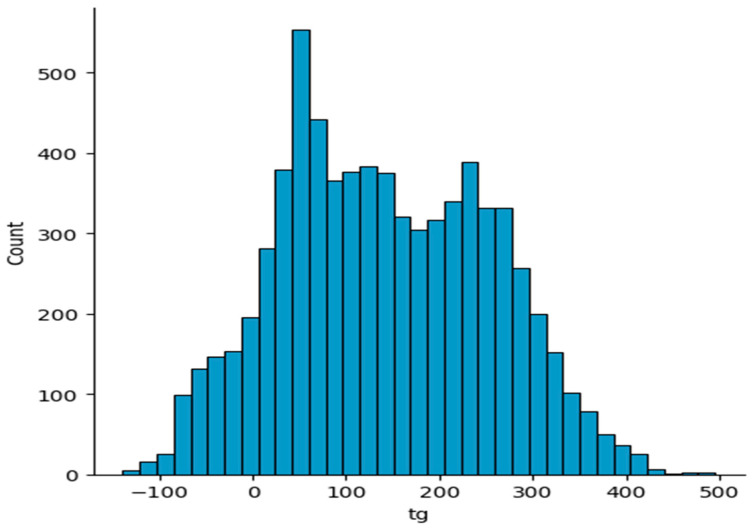
Distribution of glass transition temperatures of the polymers in the dataset.

In our work, a total of 7174 polymers exhibiting a diverse range of glass transition temperatures were selected from the reference cited as ref. [28]. The dataset consists of a blend of experimental and computed values. Experimental data give direct measurements and are generally considered more accurate. However, computed values can sometimes offer valuable insights, particularly when there are a lack of experimental data or experimental data are not available. This dataset utilizes monomers as polymer graphs to forecast the glass transition temperature. The monomer graphs possess two distinct features that indicate the points of polymerization for the monomers. The glass transition temperature of the substance refers to the specific temperature range in which the glass transition occurs. The distribution of glass transition temperatures in the polymer dataset is shown in Figure 2. The glass transition temperature exhibits a maximum value of 495.0, a minimum value of −139.0, and an average value of 141.95. The first step in running the tests was to obtain the simplified molecular input line-entry system (SMILES) notation for the polymer structures. The compounds were canonicalized using RDKit to generate uniform SMILES notations [29]. 

### 2.2. Data Preprocessing 

By minimizing the complexity of the dataset and removing unimportant or noisy attributes, we may enhance the model’s capacity to detect significant connections and patterns within the data. This technique has the potential to improve learning efficiency and boost generalization performance, ultimately leading to enhanced accuracy in real-world situations [30,31].

**Figure 3:**
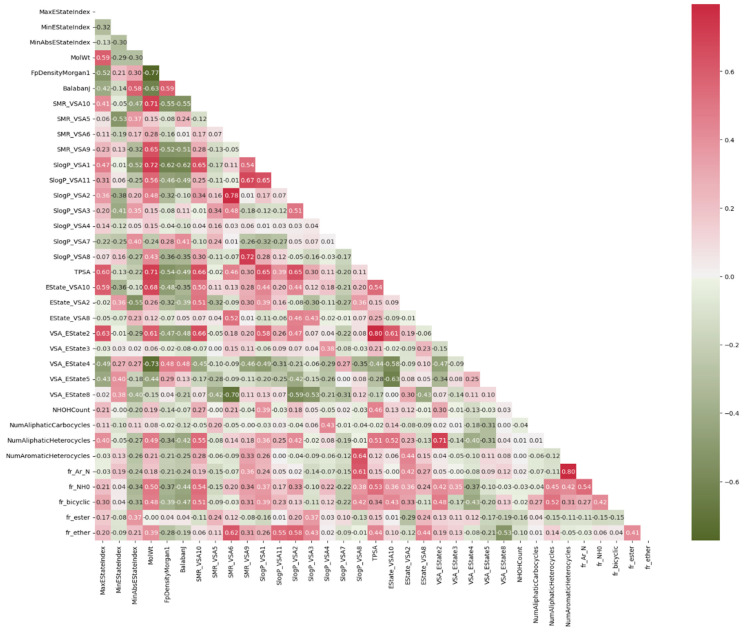
Correlation of each feature using the Pearson correlation technique.

For this reason, the Pearson correlation technique is employed first to eliminate elements that exhibit strong correlations. The outcome of this process is depicted in Figure 3. Additionally, we proceed to exclude features with a low variance from our dataset. In machine learning problems, a large amount of data has the capacity to inadvertently introduce bias into the prediction. In order to achieve equitable consideration of all variables in our models, each input variable underwent a normalization process, which involved transforming the original values into new normalized values. This transformation was carried out according to the following formula:

(1) $$Z_{i}=\frac{X_{i}-\bar{X}}{\sigma }$$

where $Z_{i}$ represents the new normalized value, $X_{i}$ represents the original value, $\bar{X}$ represents the mean of all values, and *σ* represents the standard deviation [32].

### 2.3. Polymer Representation

The utilization of polymer data for direct-training machine learning models is not feasible. Therefore, it is crucial to represent polymer structures in a way that a computer can understand. In this section, we present a qualitative approach for characterizing polymers, along with two quantitative descriptors used to analyze these materials.

The simplified molecular input line entry system (SMILES) is a widely employed method for the concise representation of a compound’s chemical structure using line notations. SMILES employs the American Standard Code for Information Interchange (ASCII) notations, which are characterized by their inherent simplicity. Computers are capable of efficiently interpreting the notations, yielding valuable parameters for machine learning models. The utility of SMILES extends beyond that of a mere connection table, as it is a language-based structure rather than a data structure. It constitutes a true language, having a limited vocabulary and set of grammar rules. It can serve as a viable alternative to quantitative structure property relationships (QSPRs). The utilization of SMILES as a parameter for quantitative structure activity relationships (QSARs) has proven to be efficient and beneficial for numerous chemical species, since it offers a concise and effective means of describing molecular structures. The level of information contained in a SMILES representation is equivalent to that of an expanded connection table. The spatial footprint of SMILES is significantly reduced due to its compact nature, often occupying 50%−70% less area than a comparable connection table [33].

Morgan fingerprints (MF) are highly effective in capturing structural similarities and are well-suited for conducting large-scale investigations. This technique is applied to detect and classify all the substructures present in a given molecule [34]. These substructures are then encoded into a bit vector, with each substructure’s presence or absence being represented by a specific bit value. The presence or absence of specific substructures is encoded by individual bits inside the bit string. By comparing the bit strings of various molecules, it is possible to assess their level of structural similarity. The feature representation is presented in a vector format, which offers flexibility for employment in different machine learning models. Morgan fingerprints are widely used in the field of cheminformatics because they are specialized in encoding chemical structures for a variety of computational uses [26].

Molecular descriptors provide a comprehensive depiction of molecular characteristics, which can enhance the accuracy of predictions but may necessitate additional processing resources. It includes both numerical and categorical descriptions of chemical compounds [35]. They are able to describe a wide range of structural, chemical, and physical properties of molecules. A vector of feature representations is derived from molecular structures to quantify physical and chemical characteristics. The inclusion of extra calculations for descriptors, such as those informed by quantum chemistry, necessitates a greater investment of labor and time compared to the utilization of the Morgan fingerprint. Prominent software libraries and tools, such as RDKit version 2022.09.5, Open Babel, and ChemPy, offer a diverse array of functions that facilitate the computation of many chemical descriptors. The use of descriptors is of utmost importance in the transformation of intricate chemical structures into numerical or categorical representations, which are then employed in machine learning models and other computer studies. Chemical compounds enable researchers to extract significant information, facilitating drug discovery and material design [27].

### 2.4. Feature Selection Method

The Morgan fingerprint and molecular descriptor exhibit a high degree of complexity, and it is important to note that not all of its elements may be significant in accurately defining a specific attribute. Indeed, the inclusion of irrelevant variables frequently results in a decreased predictive capacity. From a practical standpoint, the inclusion of huge fingerprint and descriptor dimensionalities in a system also results in increased training times. Hence, it is crucial to determine the optimal subset of the complete fingerprint and descriptor that is necessary for successfully forecasting a certain attribute. Instead of manually selecting fingerprint or descriptor components, researchers can employ various dimensionality reduction strategies to automatically choose a set of features that accurately represent a specific trait. In our study, the recursive feature elimination (RFE) algorithm is employed. The process is iterative and involves choosing attributes based on the correctness of the model. Each iteration determines the ranking score metric and eliminates low-ranking features. The recursive operation repeats until the desired number of features has been achieved. Every phase of recursive deletion calculates the accuracy metric to evaluate the model’s performance after feature removal. Analyzing how accuracy measures vary with each iteration may indicate each feature’s value to model performance. The machine learning process selected the most efficient set of attributes based on its best overall precision [1]. 

### 2.5. Feature Ranking Technique

Feature ranking is a technique used to assess the importance of characteristics in a dataset. The aim of this evaluation is to evaluate the impact of different characteristics on the predicted performance of a machine learning model. During the course of our inquiry, we employed various strategies such as information gain, Pearson correlation, and reliefF for the purpose of rating aspects. The concept of information gain (IG) is contingent upon the notion of entropy, which serves as a measure of the impurity or uncertainty inherent in a given dataset [36]. Pearson’s correlation coefficient (PCC) is computed to calculate the relationship between variables within a certain class with the aim of determining the attribute’s value [37]. The determination of a feature’s value in reliefF is achieved through a continuous process of sampling an instance and assessing the attribute’s value in relation to the nearest instances of both identical and distinct categories [38].

### 2.6. Statistical Analysis 

The Chi-square test employs the *p*-value to calculate the significance of a feature’s association with the predictor variables. A *p*-value exceeding 0.05 suggests that the attributes of the category lack correlation with the target dependent variable. Conversely, when the *p*-value falls below 0.05, it indicates a probable correlation between the category attributes and the dependent variable. The equation for χ^2^ is given below:

(2) $$\chi^{2}=\sum_{k=1}^{n}\frac{{\left(O_{k}-E_{k}\right)}^{2}}{E_{k}}$$

where the observed frequencies are denoted as $O_{k}$, the predicted frequencies are denoted as ${\;E}_{k}$, and the sample size is denoted as n [39].

### 2.7. Machine Learning Model

In our research, the decision tree, support vector machine, AdaBoost, K-nearest neighbor, extreme gradient boosting, random forest, light gradient boosting, histogram gradient boosting, and extra tree machine learning models were utilized. Their descriptions are provided below.

The decision tree [40] is a nonparametric computational approach utilized for both classification and regression applications, relying on a hierarchical tree structure. The classification and regression tree (CART) is an example of a binary tree structure that consists of a limited number of nodes, each with two child nodes at the output. The architecture is comprised of intermediate nodes that execute the test on input variables as well as terminal nodes that indicate the class labels. The tree is formed using the growing tree method, and the selection of splitting points is determined by the utilization of a greedy algorithm. The algorithm evaluates all the provided variables by considering various splits and selecting the one that maximizes the reduction in node impurity. The Gini cost function is employed to measure the purity of the nodes.

Support vector regression [41] is a supervised learning approach that is employed for the purpose of predicting discrete values. It aims to optimize the margin of tolerance by customizing the hyperplane in such a manner that minimizes the error. Linear support vector regression (SVR) is employed for a simple dataset. In the context of handling intricate data, the nonlinear support vector regression (SVR) technique utilizes kernel functions to transform the data into a higher-dimensional space, thereby enabling linear separability. Frequently employed kernel functions encompass the linear kernel, polynomial kernel, sigmoid kernel, and others. 

AdaBoost, also known as adaptive boosting [42], is an ensemble method that enhances the performance of weak estimators in order to provide a more robust and precise regressor for the purpose of process prediction. The procedure begins by applying a regressor to the original data values. Subsequently, additional instances of the regressor are fitted to the identical data, but with adjustments made to the weights of each instance based on the error of the present prediction. Moreover, it is utilized to enhance the efficacy of several machine learning methods. This approach demonstrates particular efficacy when implemented for individuals who have slower rates of learning. Ensemble algorithms are commonly employed in the domain of material science [43]. 

The K-nearest neighbor (KNN) [44] method is a statistical procedure that differs from model-based algorithms. It is versatile in its use, as it may be effectively utilized for both classification and regression tasks. In order to generate a forecast for an unknown datapoint, this algorithm identifies the K-nearest neighbors to the datapoint within the range of features. The selection of a distance metric, such as Euclidean distance, influences the definition of proximity or “closest” in a certain context. After identifying the neighbors, the algorithm proceeds to determine the average, or weighted average, of their respective goal values. The aforementioned value is the estimated forecast for the forthcoming data point [45]. 

The random forest method is a type of ensemble approach that utilizes numerous decision trees and incorporates a training procedure with a minor degree of randomization in order to enhance overall performance. The ultimate outcome of the regression or classification process is determined through the application of a weighted average or weighted vote, taking into account all predictions generated by the forest. Furthermore, the utilization of RF has the potential to offer an inherent measure for assessing the significance of individual descriptors. This capability is beneficial in the context of the logical development of polymers. The number of individual trees in our RF model is adjusted to achieve a balance between forecast accuracy and computational cost [46]. 

XGBoost [47] is a technique in ensemble learning that employs gradient boosting. This approach iteratively updates the classifier by assigning weights to the components that are not accurately categorized by the classifier. The hyperparameters necessary for constructing the XGBoost model were acquired through a grid search. It incorporates regularization algorithms that effectively mitigate the issue of overfitting. The model incorporates parameters that govern the intricacy of the individual trees as well as the overall complexity of the model. 

Hist gradient boosting regression is a technique used in gradient boosting for building quicker decision trees. The technique of binning or discretizing can significantly enhance the efficiency of the training tree models that are subsequently incorporated into an ensemble. The hist gradient boosting method utilizes its algorithm to implement the processing of input variables. Every tree incorporated into an ensemble endeavors to rectify the predicted flaws by leveraging the existing models within the ensemble [48]. 

The light gradient boosting machine (LGBM) [49] is a machine learning algorithm that leverages decision-tree techniques to address a variety of problems, including regression, classification, and other related tasks. The system has been purposefully engineered to exhibit a high efficiency, scalability, and proficiency in managing extensive datasets. Gradient-boosted trees operate by training models in a sequential manner, where each succeeding tree is trained to learn from the errors made by the prior trees. Additionally, this technique also employs the histogram-based algorithm and a leaf-wise growth strategy for the trees to enhance the efficiency of training and minimize memory consumption. One notable distinction between LGBM and other gradient boosters based on trees is the vertical growth of trees in LGBM, as opposed to the horizontal growth observed in other methods. Moreover, it has been demonstrated that LGBM exhibits a higher accuracy and efficiency compared to alternative gradient boosting methods, since it is capable of delivering more precise outcomes within a shorter timeframe [50]. 

The extra tree (ET) approach demonstrates computing efficiency and the ability to handle input vectors with high dimensions [51]. This algorithm employs the same underlying principle as the RF algorithm. Nevertheless, in order to mitigate the risk of overfitting, ET regression employs a technique where a random subset of features is utilized to train each individual base learner. RF uses the bootstrap approach to train regression trees, whereas ET utilizes the entire training set for each individual tree. In general, it is widely acknowledged that both the random forest and extra tree algorithms exhibit comparable performances. However, the extra tree method has been seen to surpass random forests in scenarios where noisy characteristics are present [52].

### 2.8. Hyperparameter Optimization Technique 

To enhance the quality of the model, many crucial hyperparameters were taken into account for tuning before conducting the evaluations. The grid search was performed in order to systematically alter the selected values. In order to enhance the efficiency of grid search implementation, the tuning procedure commenced by exploring a wide range of hyperparameter values and performing a preliminary grid search with a smaller number of times or fewer training sets. Additionally, a more focused search was conducted by increasing the number of epochs. The selection of a sequential tuning strategy was motivated by the considerable number of variables involved in the tuning process [53].

### 2.9. Shapley Additive Explanations (SHAP) 

The SHAP [54,55] framework explains the output of machine learning models by employing principles from game theory. This method quantifies the contributions of features to the predictions made by the model. DeepSHAP, Linear SHAP, TreeSHAP, and Kernel SHAP are model explanation techniques used for computing SHAP values in various types of models. The utilization of bee swarm, violin, bar, and river flow plots effectively highlights this prominent aspect. We employed bar and violin diagrams to illustrate the significance of these features. Bar graphs illustrate the impact of individual features on the predictions of a model. The features are arranged in descending order based on their highest absolute SHAP values. Violin plots are employed to visually represent control directionality across all properties.

### 2.10. Performance Evaluation Metrics 

The final step after developing a machine learning model is to evaluate its efficacy. Typical assessment procedures include the hold-out technique [56], bootstrapping sampling [57], and cross validation (CV) [58]. The objective of CV is to prevent a ML method from overfitting. The most common CV formats include leave-one-out CVs as well as k-fold CVs. Unfortunately, CV computations may involve substantial computational expenses. The prediction ability of a machine learning method is calculated by comparing the actual values to the model’s predicted values [59]. ML-computed metrics, depending on the machine learning algorithms, are used to evaluate model quality. The determination coefficient ($R^{2}$), mean absolute error (MAE), and root mean square error (RMSE) are used to measure the performance of regression algorithms [60,61]. $R^{2}$, MAE, and RMSE are described as follows:

(3) $$R^{2}=\frac{{\left[\sum_{i=1}^{t}\left(c-{\overset{´}{q}}_{e})(q_{u\left(i\right)}-{\overset{´}{q}}_{u}\right)\right]}^{2}}{\sum_{i=1}^{t}{\left(q_{e\left(i\right)}-{\overset{´}{q}}_{e}\right)}^{2}\sum_{i=1}^{t}{\left(q_{u\left(i\right)}-{\overset{´}{q}}_{u}\right)}^{2}}$$

(4) $$MAE=\frac{1}{t}\sum_{i=1}^{t}\left|q_{e(i)}-q_{u(i)}\right|$$

(5) $$RMSE=\sqrt{\frac{1}{t}\sum_{i=1}^{t}{\left|q_{e(i)}-q_{u(i)}\right|}^{2}}\;$$

where the sum of samples is *t*, the expected value is $q_{u\left(i\right)}$, the actual value is $q_{e\left(i\right)}$, the average value of all expected set is ${\overset{´}{q}}_{u}$, and the average value of all real sets is ${\overset{´}{q}}_{e}$.

## 3. Experimental Results 

In our work, we implemented a variety of machine learning regressors, including DT, SVR, AB, KNN, XGB, RF, LGB, HGB, and ETR. The experimental work was performed at the Google Colaboratory using Scikit-Learn in Python. The ten-fold cross-validation approach [62] is applied in this study to develop prediction models. The datasets are randomly split into equivalent 10 folds in the 10-fold cross-validation method. When constructing the model, nine folds are employed for training, and one is utilized for testing. This technique is repeated ten times, and then the outcomes are averaged. To validate the experiment results, various assessment metrics, such as the determination coefficient ($R^{2}$), mean absolute error (MAE), and root mean square error (RMSE), are applied. In the context of the mean absolute error (MAE) and root mean square error (RMSE) metrics, a lower value is indicative of a greater performance. Conversely, in the case of the R-squared ($R^{2}$) metric, a higher value is associated with a superior performance.

### 3.1. Finding Significantly Associated Features Using Statistical Methods 

**Figure 4:**
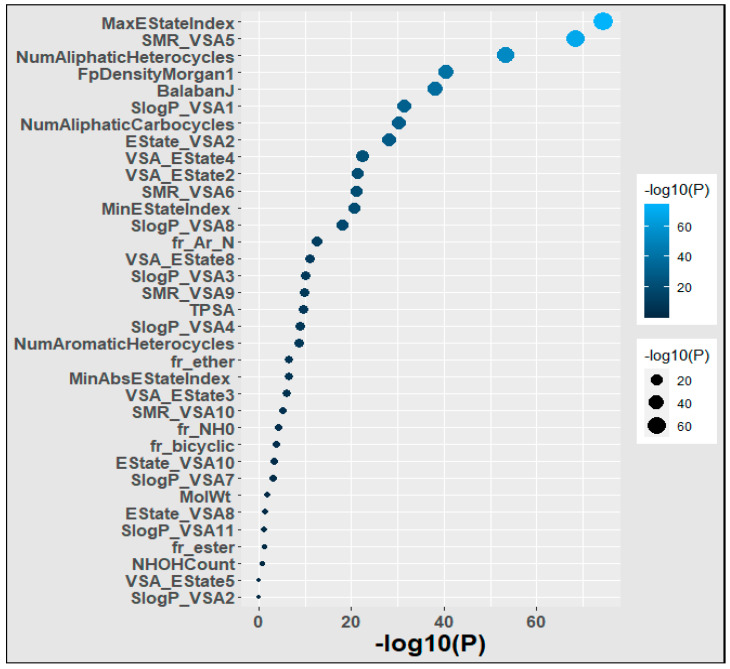
Significance of the features of the polymers. Larger and lighter bubbles represent greater significance.

We applied the Chi-square test to the polymer dataset in order to detect the most influential feature of the polymers. Our results are depicted in Figure 4. We found that MaxEStateIndex, SMR_VSA5, NumAliphaticHeterocycles, FpDensityMorgan1, BalabanJ, and SlogP_VSA1 were the most important descriptors because they exhibited a strong correlation with the goal’s property and efficiently incorporated the relevant molecular characteristics that influenced that target attribute. SlogP_VSA2, VSA_EState5, NHOHCount, fr_ester, and SlogP_VSA11 were the least important descriptors because they had little association with the targeted property and inadequately incorporated the molecular factors that affected it.

### 3.2. Prediction of Glass Transition Temperature Using Machine Learning Techniques 

The anticipated outcomes of several machine learning methodologies for the Morgan fingerprint are presented in Table 1. The XGB algorithm had the highest $R^{2}$ at 82.96%, along with the lowest MAE of 32.823 and RMSE of 46.241 when compared to other regression algorithms. RF, LGB, and HGB also demonstrated excellent performance across all the evaluation metrics. In contrast, AB exhibited the lowest $R^{2}$ at 62.71%, the highest MAE at 55.384, and the highest RMSE at 69.528. 

Table 2 demonstrates the results obtained by employing machine learning approaches with hyperparameter adjustments in the context of the Morgan fingerprinting method. The HGB model demonstrated the greatest coefficient of determination ($R^{2}$) of 83.35% and the lowest root mean square error (RMSE) of 45.719 among all the regression models. In contrast, the XGB model exhibited the lowest mean absolute error (MAE) of 32.247 when compared to the other regression models. However, the XGB model yielded the second-most favorable result. In addition, the remaining regression models, namely SVR, KNN, LGB, HGB, and RF, also yielded exceptional results. 

In Table 3, the expected outcomes of several machine learning methodologies for molecular descriptors are displayed. Compared to the other regression algorithms, the ETR algorithm had the highest $R^{2}$ at 87.83%, along with the lowest MAE of 26.243 and RMSE of 38.99. Additionally, RF, XGB, LGB, and HGB demonstrated outstanding performances across all the evaluation metrics. SVR had the lowest $R^{2}$ value of 65.88% and the highest RMSE of 65.51. In contrast, AB had the highest MAE among all the regressors at 51.453. 

**Figure 5:**
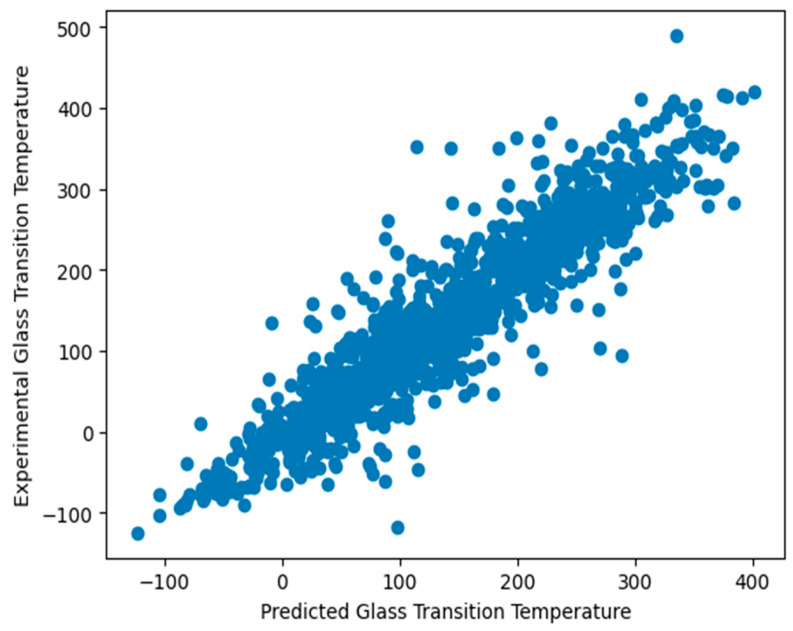
Scatterplot comparing the predicted and experimental glass transition temperatures of the polymers.

The findings achieved utilizing machine learning techniques with hyperparameter tuning using the molecular descriptor method are presented in Table 4. The ETR model had the highest $R^{2}$ value of 88.01% and the lowest MAE value of 26.186, as well as the lowest RMSE value of 38.839 when compared to all the other regression models. The SVR, KNN, XGB, RF, LGB, and HGB models produced favorable outcomes. On the other hand, AB exhibited the lowest $R^{2}$ value of 68.39%, a MAE of 50.553, and an RMSE of 63.016 compared to all the other regression models. Figure 5 shows a scatter plot of the extra tree regressor’s predicted and measured glass transition temperatures of the polymers.

### 3.3. Feature Ranking Using Machine Learning Techniques

**Figure 6:**
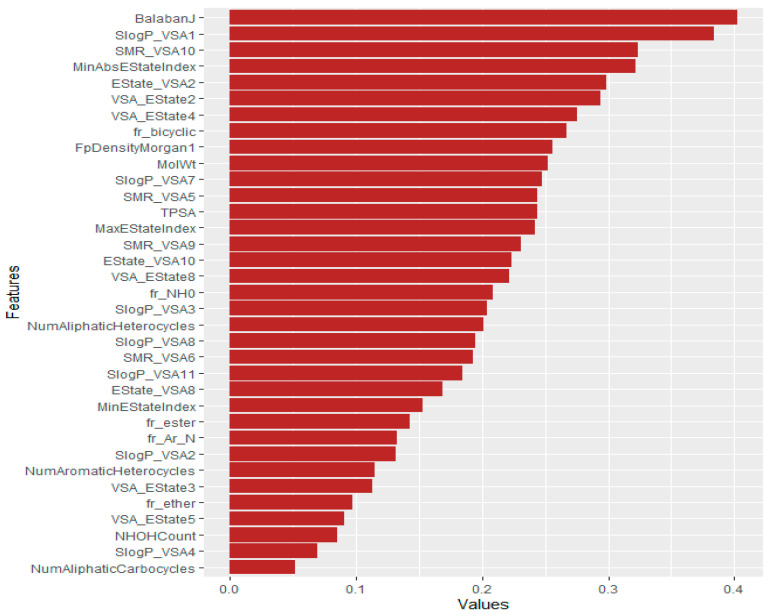
Feature ranking of polymers using machine learning techniques. Longer bars indicate the most significant features.

We also ranked the features using mutual information, Pearson correlation, and reliefF machine learning techniques. First, we calculated the feature importance value of mutual information, Pearson correlation, and reliefF. Then, the average values of these three methods were computed. The outcomes are depicted in Figure 6. In Figure 6, we analyzed the relevance of the polymer’s descriptors and identified BalabanJ as the most prominent. Other critical descriptors include SlogP_VSA1, SMR_VSA10, MinAbsEStateIndex, and Estate_VSA2.

NumAliphaticCarbocycles, SlogP_VSA4, NHOHCount, VSA_EState5, fr_ether, VSA_EState3, etc. were the least essential descriptors.

### 3.4. Analysis of the Significance of Features on Model Output

**Figure 7:**
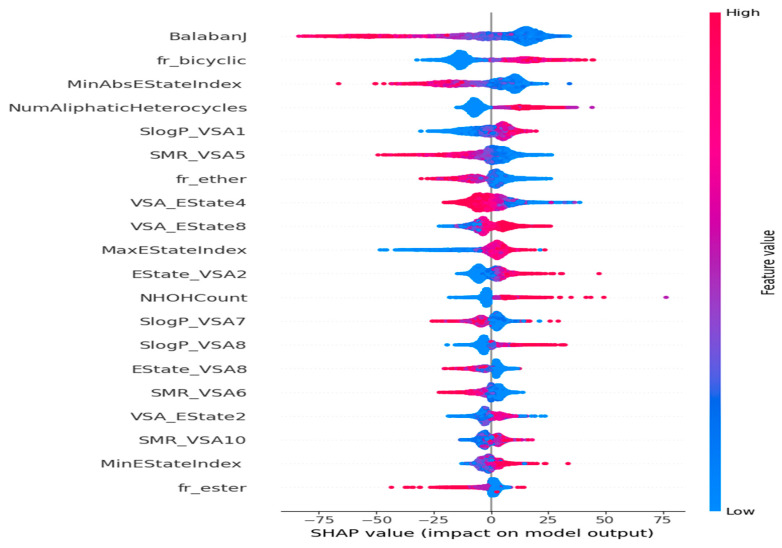
Impacts of features on the outcomes of the model evaluated using the SHAP method. The feature effects decrease in a top-to-bottom manner.

**Figure 8:**
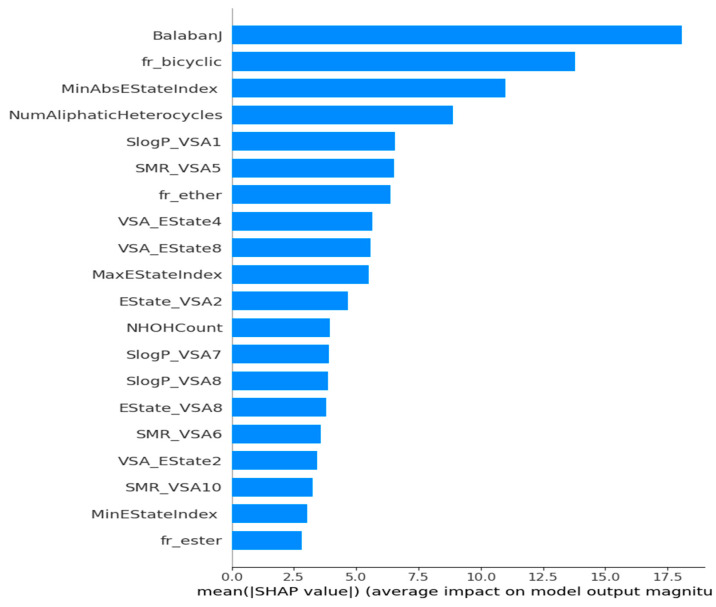
Bar chart summarizing the effects of the features on the model outcome. Longer bars represent the most important features.

The framework is specifically designed for the purpose of interpreting the outcomes of a model. One notable benefit of SHAP is its ability to accurately quantify the influence of a feature on each individual sample, providing insights into both its positive and negative impacts. The SHAP values corresponding to each molecular descriptor are presented in a ranked manner, as shown in Figure 7. This approach enabled the identification of BalabanJ, fr_bicyclic, NumAliphaticHeterocycles, and SlogP_VSA1 as the key descriptors in the model, indicating their significance in properly predicting results. The horizontal coordinate represents the magnitude of the impact on the expected value of Tg depicted in Figure 8. BalabanJ, fr_bicyclic, and MinAbsEStateIndex were the most essential descriptors. On the other hand, the descriptors fr_ester, MinEStateIndex, and SMR_VSA10 were the least significant.

Through the utilization of explainable machine learning techniques, we gained significant understanding of the internal mechanisms of the model and the individual impacts of each descriptor, ultimately improving the model’s clarity and comprehensibility. 

## 4. Discussion

The accurate estimation of the glass transition temperature of polymers is of paramount importance in the process of polymer design. After quantitatively depicting polymer data using Morgan fingerprinting and molecular descriptors, the recursive feature elimination technique was used to identify the most important features. We conducted a separate application of statistics and machine learning algorithms to the dataset of polymers. The features were assessed, and the glass transition temperature of the polymers was estimated using a machine learning technique. The hyperparameters of the machine learning model were also adjusted. Next, statistical approaches were employed to determine the significant characteristics of the polymers. The SHAP methodology was also used to demonstrate the influence of specific features on the model’s output. 

The utilization of machine learning models is prevalent in the prediction of the glass transition temperature of polymers. The ability of machine learning algorithms to identify hidden patterns within a dataset through the analysis of various features might contribute to a more inclusive comprehension. The predictions that exhibited a greater accuracy score demonstrated a level of reliability in forecasting and ensured practical relevance in real-world scenarios. In the particular case of molecular descriptors, the ETR technique yielded the highest results, with an $R^{2}\;$value of 88.01%, a MAE of 26.186, and an RMSE of 38.839. The HGB, XGB, and LGB approaches also showed excellent performances. The order of rating for the machine learning algorithms in predicting Tg, from highest to lowest performance, is as follows: ETR, LGB, XGB, HGB, SVR, RF, KNN, DT, AB. It was observed that molecular descriptors exhibit superior performance compared to Morgan fingerprints. It was found that optimizing the hyperparameters of the machine learning techniques led to an improved performance. 

The findings of our study indicate that there are several crucial and relevant features that may be used in the estimation of the glass transition temperature of polymers. The Chi-squared test revealed that the MaxEStateIndex, SMR_VSA5, NumAliphaticHeterocycles, FpDensityMorgan1, BalabanJ, and SlogP_VSA1 were the most significant features. The key attributes of the machine learning model include BalabanJ, SlogP_VSA1, SMR_VSA10, MaxEStateIndex, Estate_VSA2, and VSA_EState2. Furthermore, we identified noteworthy indicators, namely MaxEStateIndex, BalabanJ, and SlogP_VSA1, which showed identical characteristics in both the statistical association and machine learning approaches. Our work indicates that the identification of important features is adequate for the prediction of the glass transition temperature of polymers, thereby enabling the efficient design of polymers.

In the preceding study, Liu et al. [28] achieved the highest coefficient of determination $R^{2}$ value of 86.4% and the lowest root mean square error (RMSE) value of 41.2. However, our research yielded a higher coefficient of determination $R^{2}$ at 88.01%, indicating a strong relationship between the variables. Additionally, we observed a lower mean absolute error (MAE) of 26.186 and a lower root mean square error (RMSE) of 38.839, suggesting a high level of accuracy in our findings. It might be argued that the results obtained from the current effort with the identical dataset are superior to those achieved in past endeavors.

The glass transition temperature of polymers is a key material parameter in determining their mechanical behavior at room temperature. When the glass transition temperature is higher than room temperature, the polymers are flexible, like rubber, elastomers, etc. Such polymers have a large deformation capability and improved strength at a high strain rate [63,64,65,66]. Thus, these polymers are a promising candidate for energy absorption in impact engineering. When the glass transition temperature is lower than room temperature, the polymers are rigid, like polymethyl methacrylate. They also can be applied for energy absorption after microstructural modifications [67,68,69,70,71]. Therefore, this work offers an intellectual method for determining the glass transition temperature of polymers for applications as absorbent materials of mechanical energy.

## 5. Conclusions 

This study presents a machine learning framework to predict the glass transition temperature of polymers. In this study, we employed Morgan fingerprinting and molecular descriptor approaches in order to quantitatively represent polymers. Subsequently, we employed the recursive feature elimination strategy to determine significant descriptors. Next, we employed nine distinct machine learning models to analyze the aforementioned dataset, both with default and fine-tuned hyperparameter values, in order to predict the glass transition temperature. This study revealed that the extra tree technique, when applied with optimized hyperparameter values, demonstrated superior performance in the context of molecular descriptors. Additionally, we employed machine learning and statistical techniques to determine the most prominent features, resulting in the identification of MaxEStateIndex, BalabanJ, and SlogP_VSA1. The SHAP approach was utilized in our study to interpret the model. It was found that the BalabanJ, fr_bicyclic, and MinAbsEStateIndex descriptors exhibited the most substantial influence on the model. This work has the potential for further expansion in order to forecast further polymer properties, including tensile strength, Young’s modulus, toughness, elasticity, and density. It was demonstrated that this method could effectively replace empirical methodologies in the development of novel polymers with beneficial features and applications and further promote the applications of polymer-based absorbent materials in mechanical engineering.

## Figures and Tables

**Table 1:** Results of machine learning algorithms using Morgan fingerprinting.

	DT	SVR	AB	KNN	XGB	RF	LGB	HGB	ETR
$R^{2}$	0.686	0.684	0.620	0.8015	0.811	0.7981	0.7997	0.807	0.704
MAE	40.953	47.604	54.300	35.719	33.865	34.317	36.016	35.128	40.242
RMSE	62.283	62.467	68.553	49.516	48.312	49.936	49.741	48.823	60.465

**Table 2:** Results of machine learning algorithms with hyperparameter tuning using Morgan fingerprinting.

	DT	SVR	AB	KNN	XGB	RF	LGB	HGB	ETR
$R^{2}$	0.754	0.815	0.664	0.789	0.816	0.803	0.810	0.820	0.797
MAE	39.204	32.979	51.195	35.325	33.291	35.506	34.072	33.032	37.105
RMSE	55.077	47.798	64.378	51.040	47.730	49.354	48.389	47.099	50.123

**Table 3:** Results of machine learning algorithms using molecular descriptors.

	DT	SVR	AB	KNN	XGB	RF	LGB	HGB	ETR
$R^{2}$	0.690	0.652	0.679	0.805	0.841	0.850	0.857	0.851	0.867
MAE	41.668	50.165	50.172	34.615	30.922	29.898	30.325	31.018	27.960
RMSE	61.840	65.560	62.955	49.033	44.295	43.011	42.010	42.900	40.477

**Table 4:** Results of machine learning algorithms with hyperparameter tuning using molecular descriptors.

	DT	SVR	AB	KNN	XGB	RF	LGB	HGB	ETR
$R^{2}$	0.734	0.861	0.690	0.829	0.862	0.825	0.859	0.861	0.869
MAE	41.774	28.057	49.570	30.779	28.882	33.971	29.696	28.937	27.895
RMSE	57.299	41.362	61.920	45.963	41.249	46.507	41.773	41.371	40.286

## Data Availability

The data are available from the authors upon request.
